# O.4.4-7 Motives and Barriers for Leisure Time Physical Activity among rural mothers with young children

**DOI:** 10.1093/eurpub/ckad133.204

**Published:** 2023-09-11

**Authors:** Olaia Eizagirre-Sagastibeltza, Uxue Fernandez-Lasa, Oidui Usabiaga

**Affiliations:** Faculty of Education and Sport, University of the Basque Country (UPV/EHU), Vitoria-Gasteiz, Spain; Society, Sport and Physical Activity (GIKAFIT) Research Group, Department of Physical Education and Sports, Faculty of Education and Sport, University of the Basque Country (UPV/EHU), Vitoria-Gasteiz, Spain; oeizagirre003@ikasle.ehu.eus; Society, Sport and Physical Activity (GIKAFIT) Research Group, Department of Physical Education and Sports, Faculty of Education and Sport, University of the Basque Country (UPV/EHU), Vitoria-Gasteiz, Spain; oeizagirre003@ikasle.ehu.eus

## Abstract

**Purpose:**

The aim of this study was to analyse the motives and barriers for mothers with young children in rural areas to engage in leisure time physical activity (LTPA), based on the following research question: What LTPA needs and interests do mothers with young children living in rural areas of Gipuzkoa have?

**Methods:**

Four focus groups were conducted in 4 different rural districts of Gipuzkoa, in which 19 women with at least one child under 9 years of age participated. The transcripts were analysed inductively by reading them repeatedly and then organised through triangulation by the researchers from a socio-ecological perspective.

**Results:**

Regarding intrapersonal factors, because of work and informal caregiving duties, they had no time nor energy for LTPA. They felt the need to take time for themselves but put their own needs on the back burner, giving priority to the needs of their children and caregiving tasks. Moreover, they did not like being physically active, nor did they like the fact that most of users of sports facilities were men.

Concerning interpersonal factors, they enjoyed doing LTPA with their friends. However, if their friends were also mothers, they found it difficult to coincide with each other. In addition, leaving their children with someone else so that they could do LTPA, made them feel guilty. Moreover, they liked the idea of doing LTPA with their family but when they put it into practice, it did not meet their personal needs.

With respect to environmental factors, deficiencies in the facilities for LTPA physical activity in their rural municipalities, distances to municipalities with sports facilities and rugged terrain were barriers to LTPA.

**Conclusions:**

This study has helped to understand the LTPA interests and needs of the many mothers with young children living in rural areas of Gipuzkoa. These aspects are considered important to consider when developing LTPA promotion policies and may also be of interest for similar contexts.

Furthermore, these results may be important for enhancing women's well-being and quality of life through the promotion of LTPA, as well as struggling gender inequalities in leisure time for women.

